# Exploiting the genome of *Thinopyrum elongatum* to expand the gene pool of hexaploid wheat

**DOI:** 10.1007/s00122-020-03591-3

**Published:** 2020-04-20

**Authors:** Lauren Baker, Surbhi Grewal, Cai-yun Yang, Stella Hubbart-Edwards, Duncan Scholefield, Stephen Ashling, Amanda J. Burridge, Alexandra M. Przewieslik-Allen, Paul A. Wilkinson, Ian P. King, Julie King

**Affiliations:** 1 School of Biosciences, The University of Nottingham, Sutton Bonington Campus, Loughborough, Leicestershire LE12 5RD UK; 2 School of Biological Sciences, University of Bristol, Bristol, BS8 1TQ UK

## Abstract

**Key message:**

One hundred and thirty four introgressions from *Thinopyrum elongatum* have been transferred into a wheat background and were characterised using 263 SNP markers.

**Abstract:**

Species within the genus Thinopyrum have been shown to carry genetic variation for a very wide range of traits including biotic and abiotic stresses and quality. Research has shown that one of the species within this genus, *Th. elongatum*, has a close relationship with the genomes of wheat making it a highly suitable candidate to expand the gene pool of wheat. Homoeologous recombination, in the absence of the *Ph1* gene, has been exploited to transfer an estimated 134 introgressions from *Th. elongatum* into a hexaploid wheat background. The introgressions were detected and characterised using 263 single nucleotide polymorphism markers from a 35 K Axiom^®^ Wheat-Relative Genotyping Array, spread across seven linkage groups and validated using genomic in situ hybridisation. The genetic map had a total length of 187.8 cM and the average chromosome length was 26.8 cM. Comparative analyses of the genetic map of *Th. elongatum* and the physical map of hexaploid wheat confirmed previous work that indicated good synteny at the macro-level, although *Th. elongatum* does not contain the 4A/5A/7B translocation found in wheat.

**Electronic supplementary material:**

The online version of this article (10.1007/s00122-020-03591-3) contains supplementary material, which is available to authorized users.

## Introduction

Modern hexaploid wheat (*Triticum aestivum*) is a staple food crop that contributes ~ 20% of global daily dietary calories (Reynolds et al. 2012). By 2050, the global population is predicted to exceed 9.7 billion, and global wheat demand is predicted to reach 900 million tonnes (Charmet 2011; FAOSTAT 2018). However, current global wheat production is only 749 million tonnes (achieved in 2016) (FAOSTAT 2017). The annual yield percentage increase, which for the last decade has averaged 0.9% globally, will need to increase to ~ 2.4%. In Europe, however, yield increases have steadily plateaued to just 0.1% (Ray et al. 2013).

Wheat, which evolved circa 8000 to 10,000 years ago, has been through a significant genetic bottle neck due to its monophyletic or diphyletic evolution and subsequent domestication (Shewry 2009). Intensive selection pressure applied by centuries of farming has also eroded the variation within the wheat gene pool, reducing the level of genetic variation available in breeding programmes for the production of elite cultivars. Emerging diseases and climate change are further impacting wheat yields (Lobell et al. 2011; Curtis and Halford 2014; Price et al. 2014). It is thus essential to increase the genetic diversity available for breeders for producing new elite cultivars of wheat that are climate change ready (Dempewolf et al. 2014).

The wild relatives of wheat represent a vast and underutilised source of genetic variation for virtually all agronomic traits of interest. Interspecific crossing with wheat’s wild relatives has repeatedly been shown to successfully transfer traits of interest (e.g. Ayala-Navarrete et al. 2007; Riar et al. 2012; Ceoloni et al. 2017).

*Th. elongatum* belongs to the genus Thinopyrum, which was segregated from the much larger genus *Elytrigia* in the late twentieth century, alongside the genera *Lophopyrum* and *Trichopryum* (Baum and Johnson 2018). Diploid (2*n* = 2*x* = 14:EE), tetraploid (2*n* = 4*x* = 28), hexaploid (2*n* = 6*x* = 42) and decaploid accessions (2*n* = 10*x* = 70) of *Th. elongatum* have been identified (Chen et al. 2013; Guo et al. 2016; Mao et al. 2010). However, the literature contains considerable confusion in distinguishing between decaploid *Th. elongatum* and another of the decaploid Thinopyrum species, *Th. ponticum.* Previously, these two species were frequently placed under the same name, *Agropyrum elongatum* (Shepherd and Islam 1988; Li et al. 2017). *Th. ponticum* has been described with three diverging genomic constitutions: E^b^E^b^E^b^E^b^E^b^E^b^E^b^E^b^E^b^E^b^ (Arterburn et al. 2011), JJJJJJJ^S^J^S^J^S^J^S^ (Chen et al. 1998) and E^e^E^e^E^b^E^b^E^x^E^x^StStStSt (Zhang et al. 1996), where the E^b^/E^e^/E^x^/J genome is derived from the closely related genomes *Th. bessarabicum*/*Th. elongatum* (diploid) and the St genome from *Pseudoroegneria strigosa.* Research has shown a range of *Thinopyrum* species carry genetic variation for a range of agronomically important traits including salinity tolerance (Dvorák et al. 1988; Colmer et al. 2006), perennial growth habit (Lammer et al. 2004), water logging tolerance (Taeb et al. 1993), improved photosynthetic capacity (Reynolds et al. 2001), resistance to a wide range of diseases (Friebe et al. 1996; Zhang et al. 2009; Fu et al. 2012; Li et al. 2017) and improved flour quality (Tanaka et al. 2017). Research has also shown a close relationship between the genomes of wheat and *Th. elongatum*, suggesting it is a highly suitable candidate to expand the gene pool of wheat (Liu et al. 2007).

This paper describes the identification and characterisation of a series of introgression lines generated between hexaploid wheat and *Th. elongatum* using genomic in situ hybridisation (GISH) and an Axiom^®^ Wheat-Relative SNP Genotyping Array.

## Materials and methods

### Generation of introgressions

**Fig. 1:**
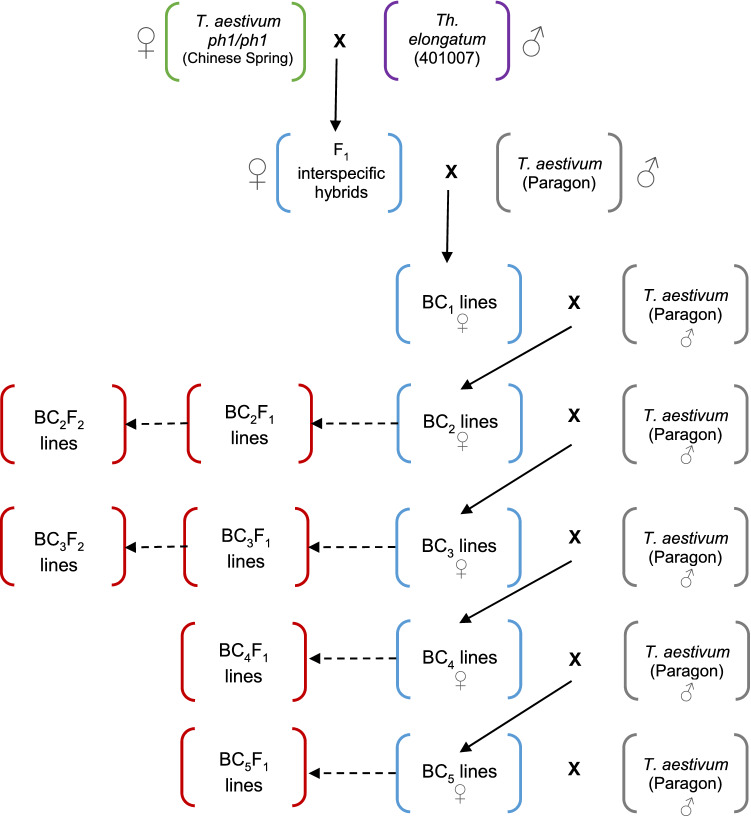
An overview of the wheat/*Th. elongatum* breeding programme showing the production of each generation via crossing (whole arrow) or via self-fertilisation (dashed arrow). Red boxes represent lines produced from self-fertilisation of the previous generation. Blue boxes represent lines produced via crossing

*Th. elongatum,* accession 401007 (2*n* = 10*x* = 70 (+4), seed obtained from the United States Department of Agriculture) was used to pollinate hexaploid wheat *ph1/ph1* mutant (cv. Chinese Spring) (Fig. 1). The resulting F_1_ hybrids were then pollinated using wheat *Ph1/Ph1* (cv. Paragon) to produce backcrossed lines (BC_1_ generation). Both of the wheat genotypes were obtained from the Germplasm Resource Unit (GRU), John Innes Centre. Further rounds of backcrossing using *Ph1/Ph1* wheat as the pollen donor produced BC_2_, BC_3_, BC_4_ and BC_5_ populations with self-fertilised lines also produced at each generation after the BC_2_ generation (Fig. 1).

### Detection and characterisation of introgressions

#### Genotyping and genetic map construction

**Fig. 2:**
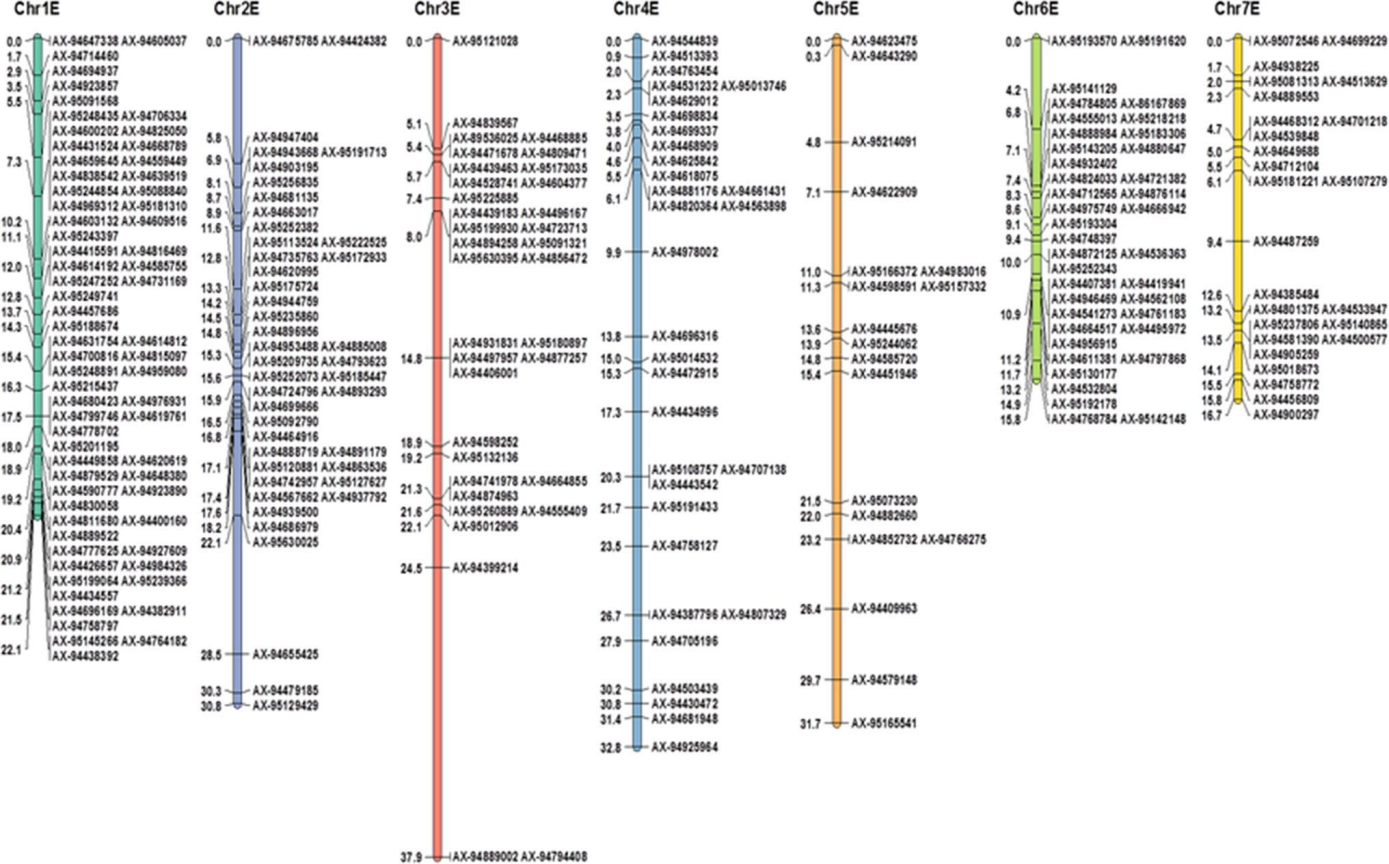
Genetic linkage map of *Th. elongatum* showing the 263 Affymetrix SNP markers spread across all seven linkage groups. SNP marker names and calculated cM distances for each group are shown. Map created using MapChart 2.3 (Voorrips 2002)

**Fig. 3:**
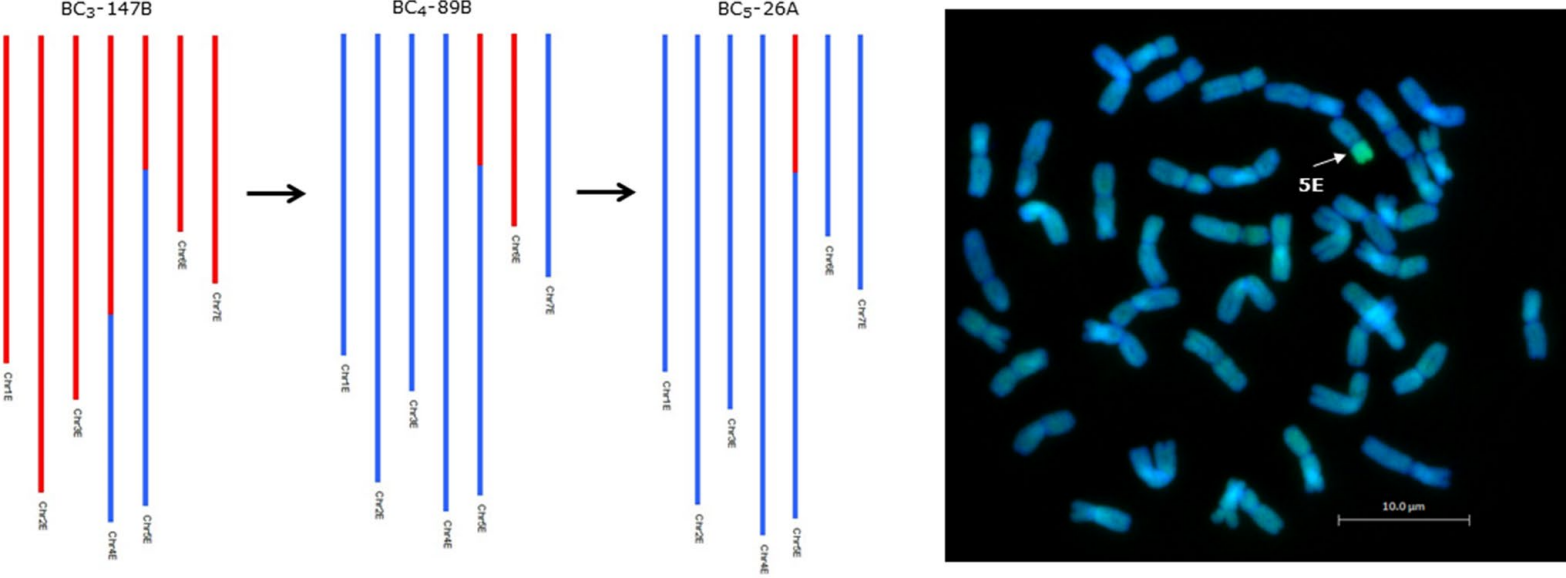
A progression of GGT genotypes (red represents *Th. elongatum* chromatin and blue represents wheat chromatin) for a BC_3_ parent, one of its BC_4_ offspring and a subsequent BC_5_ offspring. The GISH image shows a metaphase spread of BC_5_-26A and shows the introgression detected via the SNP markers (white arrow). The SNP markers enable the introgression to be identified as a linkage group 5E segment

**Fig. 4:**
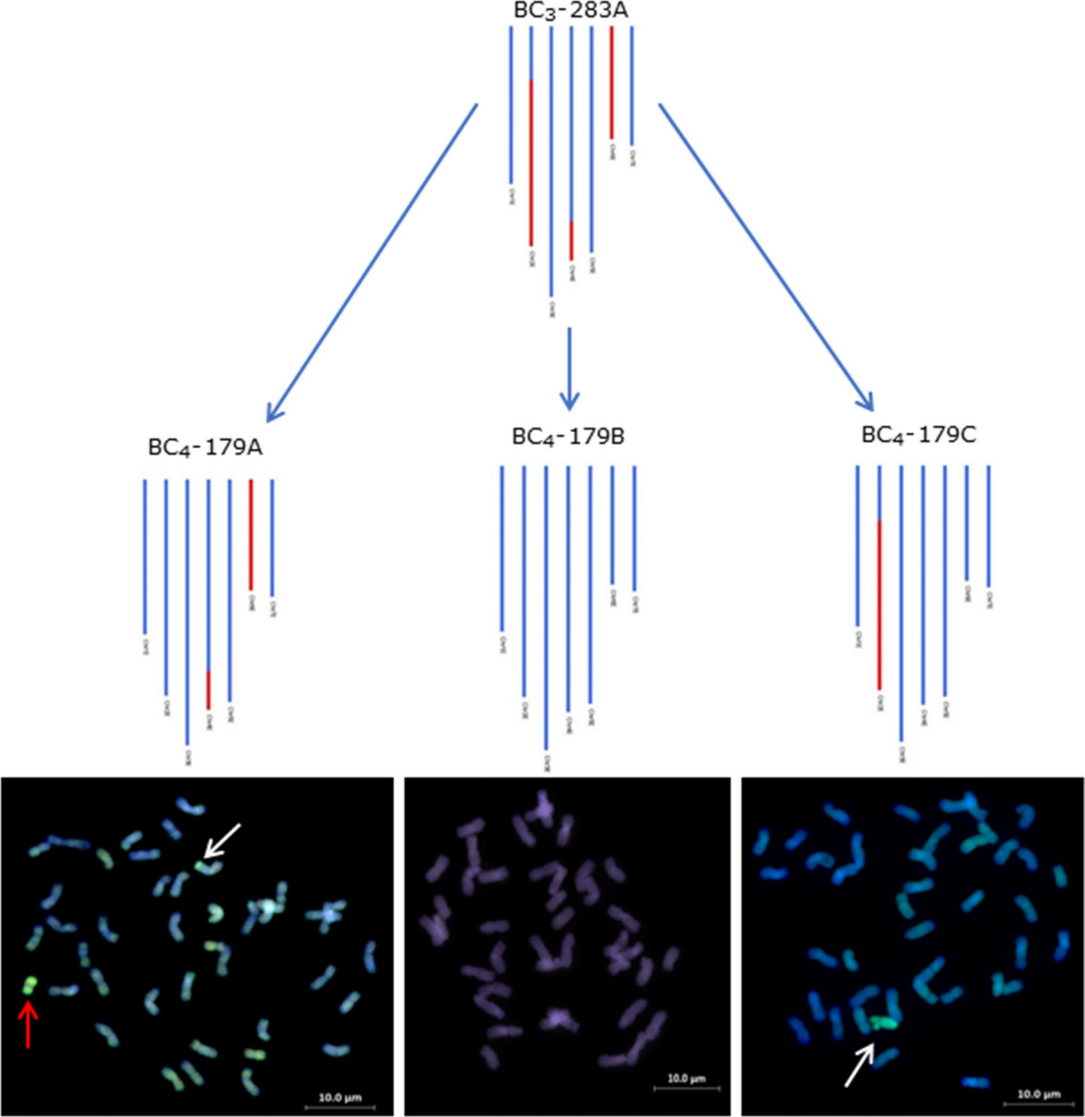
GGT genotypes for a BC_3_ plant and three BC_4_ offspring, all confirmed using GISH below their respective genotype and showing the segregation of segments in a family as the breeding programme progresses. In the GGT images, red represents *Th. elongatum* chromatin and blue represents wheat chromatin. In the GISH images, all introgressions are indicated using a white arrow, whole *Th. elongatum* chromosomes are indicated using a red arrow

Three hundred and thirty individuals from the BC_1_ to BC_5_ backcross generations, 17 Chinese Spring-*Th. elongatum* addition lines (obtained from the GRU) and three replicates of each parental line were genotyped using the Axiom^®^ Wheat-Relative Genotyping Array as described in King et al. (2017). All SNPs incorporated in the array show polymorphisms between wheat and ten wild relatives and were selected from the Axiom^®^ 820 K array (Wilkinson et al. 2012, 2016; Winfield et al. 2016). Allele calling was carried out as described by King et al. (2017). SNP markers from the categories Poly High Resolution (PHR) and Call Rate Below Threshold (CRBT) were selected for further analysis. Markers were only used in further analysis if polymorphic and co-dominant between the three replicates of wheat and *Th. elongatum*. Flapjack™ (Milne et al. 2010) was used to visualise the genotypes, and markers were removed from further analysis if they showed no call, were heterozygous for either parent or if they showed no polymorphism between the parents. Remaining markers were then analysed in JoinMap^®^ 4.0 (van Ooijen 2011) using the Haldane map function (Haldane 1919), a LOD score of 20 and a recombination frequency of 0.1. The seven highest ranking linkage groups were assigned to a chromosome group using chromosome locations for each marker described in the Axiom^®^ Wheat HD Genotyping Array (Winfield et al. 2016). Markers showing more than 20% erroneous calls were removed and markers at the same genetic position were ordered by their physical positions on the wheat reference genome, found by performing a BLAST analysis of the marker sequences against the wheat reference sequence RefSeq v1.0 (Alaux et al. 2018; International Wheat Genome Sequencing Consortium [IWGSC] et al. 2018) and obtaining the best BLAST hit from each of the three genomes of wheat, where available. Linkage group data was used to produce a genetic map using MapChart 2.3 (Voorrips 2002) (Fig. 2) and genotypes for individual lines were visualised using Graphical GenoTypes 2.0 (GGT; van Berloo 2008) (Figs. 3, 4).

#### Synteny analysis

**Fig. 5:**
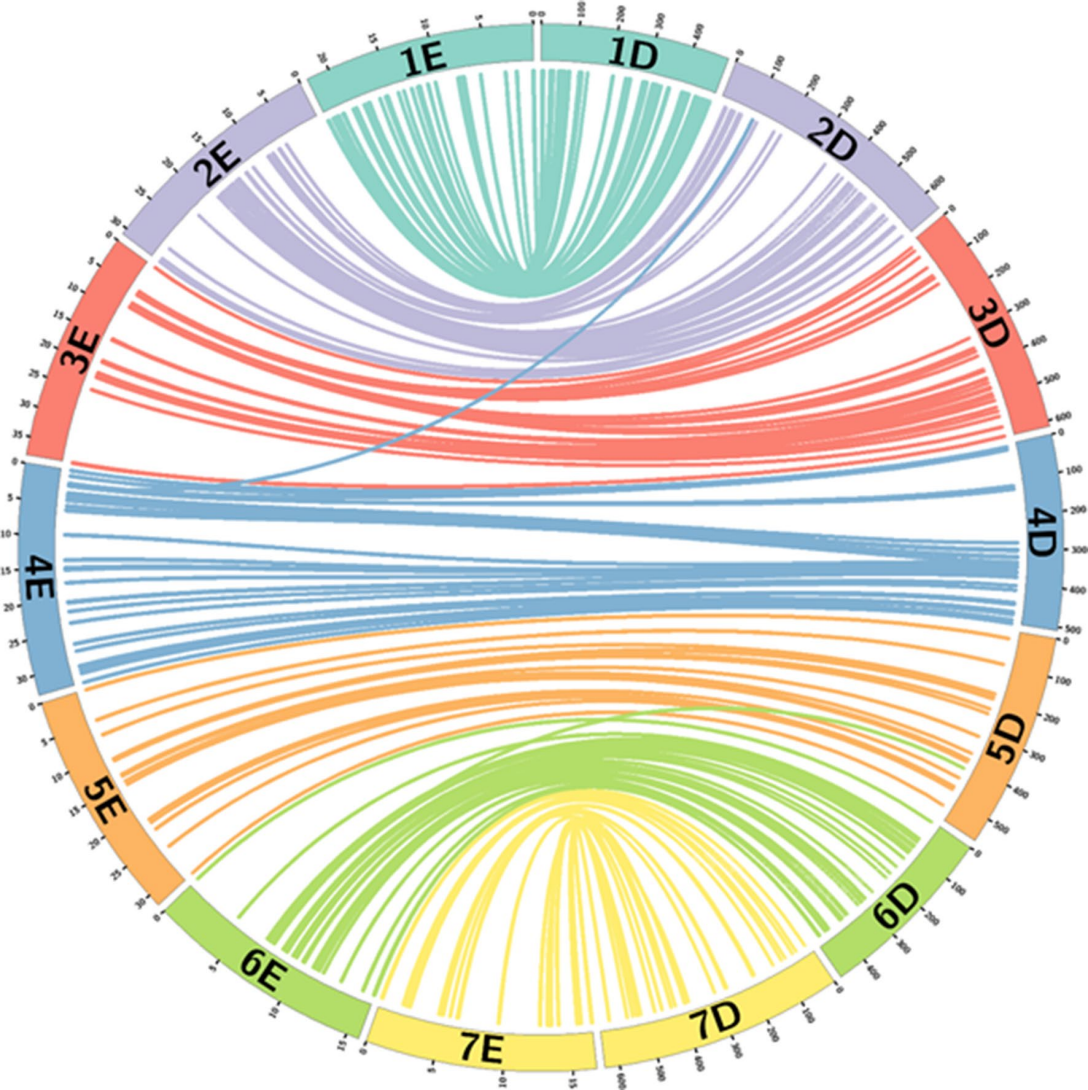
Graphical representation made using Circos showing the introgression sizes in panel plants from the wheat/*Th. elongatum* breeding programme. Each band in each linkage group represents a different individual line. Lines selected may contain whole chromosomes in other linkage groups. A total of 48 plants make up the panel

Synteny between wheat and *Th. elongatum* was analysed using the 263 SNP markers selected for the genotyping analysis. The sequences of the mapped markers were used in a BLAST search (e-value cut-off of 1e−05) against the wheat genome IWGSC RefSeq v1.0 (Alaux et al. 2018; IWGSC et al. 2018) to obtain the corresponding physical positions of the top hit in the A, B and D genomes of wheat Supplementary Table S1). The results were visualised using Circos (v. 0.67; Krzywinski et al. 2009) to show synteny between the genetic position in cM for *Th. elongatum* and the corresponding physical positions on the D genome of wheat (Fig. 5). Some markers showed the same score for the top hit for more than one genome.

#### Cytogenetic analysis

Root tips, collected from germinated seeds, were treated with nitrous oxide gas at a pressure of 10 bar for 2 h, fixed in 90% acetic acid, washed with water and then digested in 20 μl of 1% pectolyase Y23 and 2% cellulose Onozuka R-10 (Yakult Pharmaceutical, Tokyo) enzyme solution at 37 °C for 55 min (adapted from Kato et al. 2004). Samples were crushed in 70% ethanol and the cells collected via centrifugation at 2.5 g for 1 min before being briefly dried and re-suspended on ice in 25 μl of 100% acetic acid. Cell suspensions were dropped onto glass slides (7 μl per slide).

Single colour genomic in situ hybridisation (sc-GISH) was carried out as described in (King et al. 2017 and 2018; Grewal et al. 2018a) using total genomic DNA from *Th. elongatum* labelled by nick translation with Chroma Tide Alexa Fluor 488-5-dUTP (Invitrogen; C11397). Genomic DNA from *T. aestivum* (cv. Chinese Spring) was fragmented to 300-500 bp and used as blocking DNA in a ratio of 1:20 probe: blocking DNA.

For multi-colour GISH (mc-GISH) of wheat/*Th. elongatum* introgression lines, total genomic DNAs from *Triticum urartu*, *Aegilops tauschii* and *Th. elongatum* were labelled by nick translation with Chroma Tide Alexa Fluor 488-5-dUTP (Invitrogen; C11397), Chroma Tide Alexa Fluor 594-5-dUTP (Invitogen; C11400) and Chroma Tide Alexa Fluor 546-14-dUTP (Invitrogen; C11401), respectively. Slides selected from the sc-GISH analysis were then re-probed with labelled DNA from *T. urartu* (100 ng), *Th. elongatum* (100 ng), *Ae. tauschii* (200 ng) and fragmented DNA of *Ae. speltoides* (4000 ng) as blocking DNA in a ratio of 1:1:2:40.

Multi-colour GISH was also carried out on *Th. elongatum* accession 401007. Total genomic DNAs from *Th. bessarabicum* and *P. strigosa* were labelled by nick translation with Chroma Tide Alexa Fluor 488-5-dUTP and Chroma Tide Alexa Fluor 594-5-dUTP, respectively. Slides were probed with labelled DNA from *Th. bessarabicum* (100 ng), *P. strigosa* (100 ng) and genomic DNA of Chinese Spring (4000 ng), fragmented to 300-500 bp, as blocking DNA in a ratio of 1:1:40.

All sc-GISH and mc-GISH slides were counterstained with Vectashield mounting medium containing 4′,6-diamidino-2-phenylindole,dihydrochloride (DAPI) and analysed using a Zeiss Axio Imager.Z2 upright epifluorescence microscope (Carl Zeiss Ltd, Germany) with filters for DAPI and Alexa Fluor 488, Alexa Fluor 594 and Alexa Fluor 546. A Metasystems Coolcube 1 m CCD camera was used to capture images which were analysed using Metafer (automated metaphase image capture) and ISIS (image processing) software (Metasystems GmbH, Germany).

## Results

### GISH analysis of Thinopyrum elongatum

**Fig. 6:**
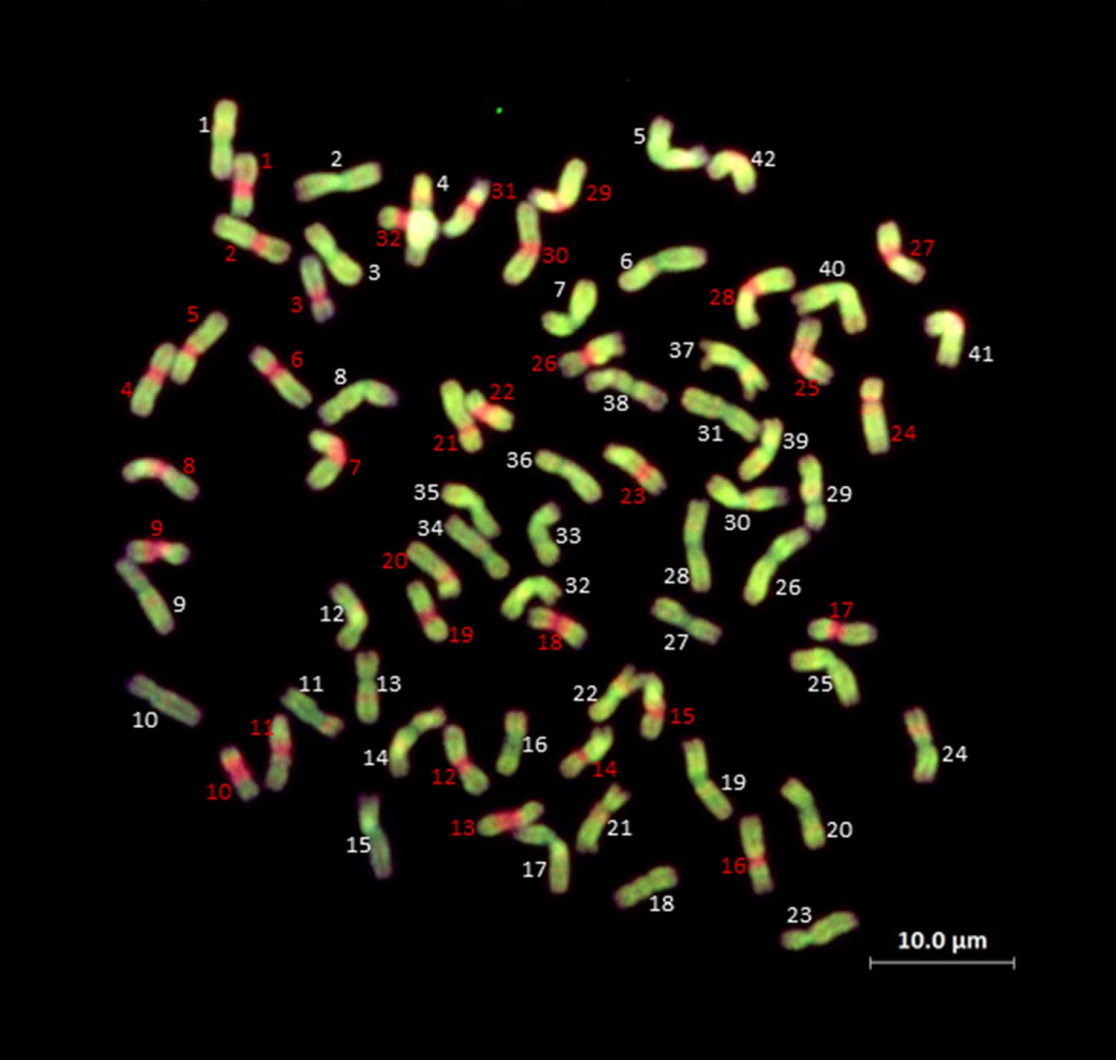
Multi-colour GISH of a metaphase spread of *Th. elongatum* accession 401007 probed with total genomic DNAs from *Th. bessarabicum* (green) and *P. strigosa* (red)

The decaploid accession used in this study (accession 401007) carried 74 chromosomes (Fig. 6). When the parental *Th. elongatum* was analysed with mc-GISH, large blocks of the St genome were observed at the centromeres of 32 chromosomes, while the remaining 42 chromosomes either did not carry any DNA from the St genome or a very faint non-centromric St genome fluorescence (Fig. 6).

### Production of wheat/Th. elongatum introgression lines

The number of seeds sown, germination success, heads crossed, cross-fertility and seed set for each generation is summarised in Table 1. In total, 29 crosses were made between *Th. elongatum* and wheat homozygous for *ph1* deletion resulting in the production of 222 F_1_ seed. The lowest germination rate was shown by the F_1_ seeds (57%) while the highest rate was shown by the BC_1_ (91%). In contrast, the BC_1_ showed the lowest cross-fertility (56%) and the lowest number of seeds set per cross (2.5) while the highest fertility was shown by the F_1_ (88%).

**Table 1:** Number of seed produced and germinated in relation to the number of crosses carried out for each generation of the introgression programme for *Th. elongatum* into wheat

	Wheat x *Th. elongatum*	F_1_	BC_1_	BC_2_	BC_3_	BC_4_	BC_5_	Totals
Number of seed sown	NA	75	88	158	227	128	112	788
Germination rate (%)	NA	57	91	82	67	84	80	–
Crosses made	29	178	633	709	208	132	–	1709
Cross-fertility (%)	66	88	56	77	66	87	–	–
Seeds/cross	1.5	5.6	2.5	5.3	4.2	7.1	–	–
% selfed heads producing seed	0	77	73	84	92	93	100	–

### Genotyping and genetic map construction

Initial genotyping analysis identified 1594 polymorphic markers between wheat and *Th. elongatum* from the 35 K Axiom^®^ Wheat-Relative Genotyping Array that were either PHR or CRBT SNP markers with good cluster resolution. Sample call rate ranged from 83.7 to 99.6% with an average of 98.3% for the 356 samples genotyped (330 backcross lines, 17 addition lines and 9 parental replicates) and the lowest call rates were obtained for the three *Th. elongatum* samples with an average of 87.3%. A total of 497 erroneous markers were removed after analysis with Flapjack™ and a further 834 markers were removed as they showed unique or inconsistent patterns of segregation. The remaining 263 SNP markers were genetically mapped to seven linkage groups using JoinMap^®^ 4.0 (Table 2) with the highest number of SNP markers mapping to linkage group 1 (26%) and the lowest to linkage group 5 (7%). The genetic linkage map of *Th. elongatum* (Fig. 2) had a total map length of 187.8 cM and an average chromosome length of 26.8 cM. From the genetic linkage maps, an estimated 134 wheat/*Th. elongatum* introgressions were generated. Plants from different generations were used for the genotyping, and thus, while the linkage map allowed the identification and characterisation of the introgressed segments and tracking through the backcross generations (Fig. 3), the cM distances need to be treated with considerable caution.

**Table 2:** Number of SNP markers polymorphic between wheat and *Th. elongatum* mapped onto the genetic map of *Th. elongatum*, the cM distance of each linkage group and the number of recombination events detected in each linkage group

Linkage group	1	2	3	4	5	6	7
Number of SNP markers	68	44	35	32	19	39	26
Linkage group cM length	22.08	30.83	37.88	32.83	31.74	15.80	16.67
Number of recombination events detected	23	25	14	24	16	16	16

### Detection of introgressions using GISH

**Fig. 7:**
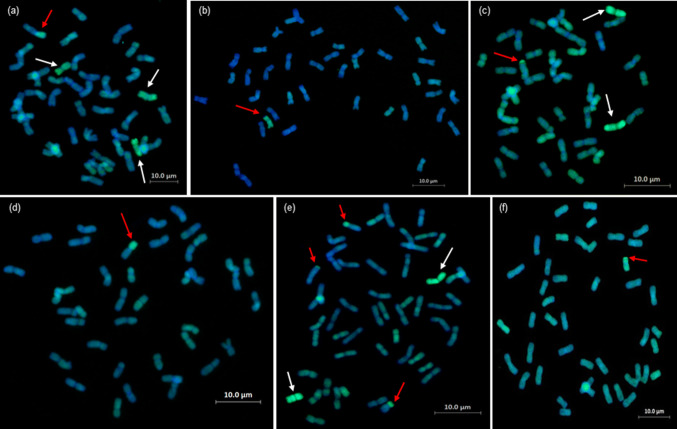
GISH images from lines from different families in the breeding programme. **a** BC_4_-118E, **b** BC_4_F_1_-101D, **c** BC_4_-183A, **d** BC_4_-172C, **e** BC_4_F_1_-90D and **f** BC_3_-670C. *Th. elongatum* chromatin is shown as green and wheat chromatin is shown as blue. All introgressions are indicated using a red arrow and whole *Th. elongatum* chromosomes are indicated using a white arrow

**Fig. 8:**
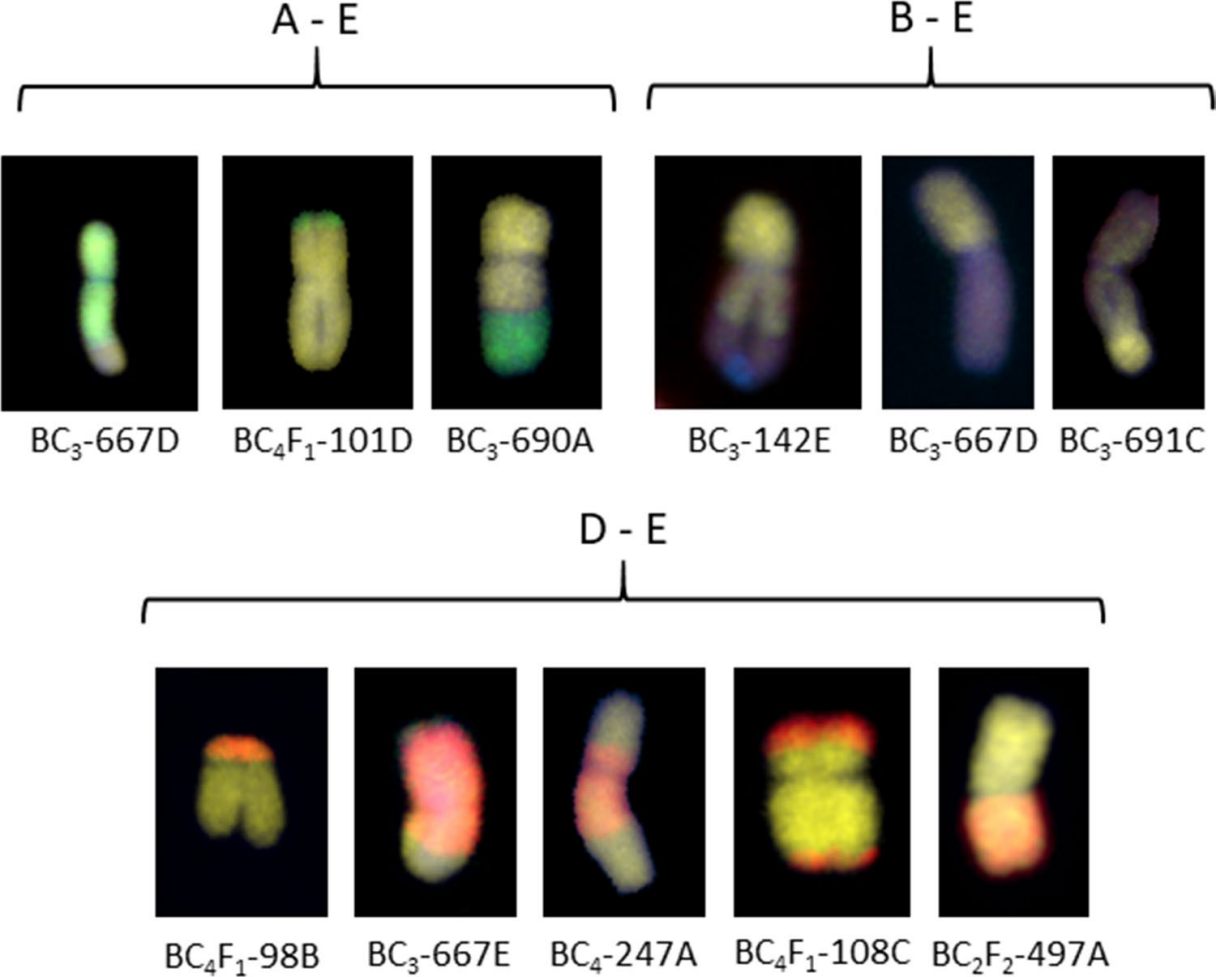
Mc-GISH results showing 11 interspecific chromosomes with the recombination events at different locations. In all images, the yellow represents *Th. elongatum* chromatin (E genome). In the A-E grouping, the green represents *T. urartu* chromatin (A genome), in the B-E grouping, the blue represents *Ae. speltoides* chromatin (blue) and in the D-E grouping, the red represents *Ae. tauschii* (D genome)

Lines analysed by sc-GISH confirmed the presence of high numbers of both whole *Th. elongatum* chromosomes and wheat/*Th. elongatum* introgressions revealed by genotyping (Fig. 7). Thus, the sc-GISH was also used to validate the introgressions identified by genotyping. In the lines analysed with both genotyping and GISH, the number of whole chromosomes and introgressions detected was the same (Figs. 3 and 4). Mc-GISH showed that recombination had taken place between *Th. elongatum* and all three genomes of wheat (Fig. 8) (E with A = 13, E with B = 11 and E with D = 4) and that the recombination events were not localised in distal regions of the chromosomes (Fig. 8).

### Synteny

Figure 5 shows the syntenic relationship between the D genome of wheat and the E genome of *Th. elongatum*. The BLAST results of the SNP markers showed that 89.4%, 92.4% and 94.5% of the markers had a significant BLAST hit on the A, B and D genomes of wheat, respectively, with 25.9%, 41.1% and 39.9% of the markers having an overall top hit on the A, B and D genomes of wheat, respectively (Supplementary Table S1). The D genome was thus selected for syntenic analysis as it was shown to have the highest number of significant BLAST hits for the markers on the *Th. elongatum* genetic map (Fig. 2).

## Discussion

The genus Thinopyrum has the potential to significantly increase the level of genetic variation available for wheat improvement., e.g. salt tolerance (King et al. 1997; Colmer et al. 2006), Fusarium head blight (Oliver et al. 2006; Ceoloni et al. 2017), increased biomass and seed number (Reynolds et al. 2001). Much of the work previously reported on *Th. elongatum* has been carried out on the diploid (2*x* = 2*n* = 14; EE). However, the species contains accessions at different ploidy levels including decaploid (Chen et al. 2013; Guo et al. 2016; Mao et al. 2010). Mc-GISH observations of the *Th. elongatum* 401007 accession indicate that the genome is composed of DNA sequences derived from at least two genomes, i.e. *Th. bessarabicum* and *P. strigosa*, and that considerable recombination/translocation has occurred between the two genomes in the past. It would also suggest that the correct genome composition of the accession under study here (401007) is EEEEEEE^St^E^St^E^St^E^St^ or JJJJJJJ^St^J^St^J^St^J^St^. This configuration was also found in *Th. ponticum* by Kruppa and Molnár-Lang (2016) again suggesting a close relationship between *Th. elongatum* 401007 and *Th. ponticum*.

In this work, introgressions were generated in the gametes of interspecific wheat/*Th. elongatum* hybrids which lacked the wild type *Ph1* locus, which normally restricts recombination to homologous chromosomes. In addition, the F_1_ hybrids only carried the haploid chromosome complement of the genomes of wheat and *Th. elongatum*. Thus, the A, B and D genome chromosomes could only undergo homoeologous recombination, i.e. with homoeologous wheat or *Th. elongatum* chromosomes. This strategy was employed in a direct attempt to increase the frequency of homoeologous recombination and hence introgression in the gametes of the F_1_ hybrids.

The germination rate (Table 1) of the F_1_ interspecific hybrids (57%), although the lowest of the generations in this crossing programme, was higher than that seen in F_1_ interspecific hybrids between wheat and other wild relatives, e.g. *Amblyopyrum muticum* (28.6%) and *Ae. speltoides* (15%) (King et al. 2017, 2018).

The rate of fertility of the F_1_ hybrids was also surprisingly high at 88% (Table 1). In crossing programmes with other wild relatives, the fertility of the F_1_ interspecific hybrids has been observed to be considerably lower, e.g. 1.6% for *Th. bessarabicum*, 16% with *Am. muticum*, 21% with *T. urartu* and 29% with *Ae. speltoides* (Grewal et al. 2018a, b; King et al. 2017, 2018). In our hands, the low fertility of F_1_ hybrids, although they frequently result in the generation of high numbers of introgressions, remains the rate limiting step with many of the wild relatives of wheat. The lower rate of fertility normally observed in wheat/wild relative F_1_ hybrids presumably results from the fact that they are essentially haploid for the genomes of wheat and those of the wild relative resulting in substantial chromosome pairing failure at meiosis leading to the generation of unbalanced gametes which as a result are often unviable.

The higher rate of fertility in the wheat/*Th. elongatum* F_1_ hybrids might be attributable to the close synteny between the E and E^st^ genomes of *Th. elongatum* and also between these genomes and the D genome of wheat (Liu et al. 2007; Wang, 2011; Hu et al. 2012), resulting in a level of chromosome pairing during meiosis that reduces the frequency of unbalanced gametes. This could also explain why the F_1_ interspecific hybrids between wheat and *Th. elongatum* show male fertility. In contrast, F_1_ hybrids between wheat and *Am muticum* (King et al. 2017), *Ae. speltoides* (King et al. 2018), *Th. bessarabicum* (Grewal et al. 2018a), *T. urartu* (Grewal et al. 2018b) and *T. timopheevii* (Devi et al. 2019) all show complete male sterility. Some of the GISH results obtained here, however, are not fully supportive of the D genome similarity (see below). The fertility rate then dropped in the BC_1_ generation.

In this work, we have utilised the Axiom^®^ Wheat-Relative Genotyping Array for the characterisation of the wheat/*Th. elongatum* introgression lines. This array was designed to contain SNPs polymorphic between wheat and all ten wild relatives under study at the Nottingham BBSRC Wheat Research Centre and thus allowed the genotyping of introgression lines from different wild relatives on the same 384 array. However, many other markers have been designed to characterise *Th. elongatum* introgressions in a wheat background including microsatellites (SSRs), expressed sequence tags (ESTs), PCR-based landmark unique genes (PLUGs), sequence-characterised amplified regions (SCARs), conserved orthologous set (COS) markers and SNPs (Hu et al. 2012; Chen et al. 2015; Dong et al. 2017; Gaál et al. 2018).

The level of interspecific recombination in the gametes of the F_1_ hybrids was such that it was possible to generate 7 discrete genetic linkage groups of *Th. elongatum* (Fig. 2), from which it could be estimated that 134 introgressions, covering the whole genome of *Th. elongatum*, had been generated. (There was no evidence from the genetic mapping or GISH of any further recombination in later generations.) The genetic map also allowed the characterisation and tracking of the introgressions through the backcross generations (Figs. 3, 4). In order to validate the genotyping data obtained, lines were also analysed via GISH. In each case, the presence, number and size of introgressions predicted via genotyping was confirmed via GISH. The production of only 7 linkage groups would be considered an unexpected observation from an allopolyploid, i.e. 2*n* = 10*x* = 70 + 4; EEEEEEE^St^E^St^E^St^E^St^. However, this observation could be explained by the high level of recombination/translocation that has occurred between the two genomes of *Th. bessarabicum* and *P. strigosa* in the past. Alternatively, the assembly of only 7 linkage groups may have resulted from the relatively low numbers of *Th. elongatum* polymorphic SNPs generated that could be used to identify introgressions as compared to similar work undertaken in other species, e.g. (King et al. 2017, 2018; Grewal et al. 2018a, b). The generation of a SNP between a wild relative and wheat requires that a base change is identified in the genome(s) of the species in question relative to the equivalent sequences in the three genomes of wheat. With diploid wild relatives, the identification of polymorphic SNPs is a relatively straight forward process, i.e. a single change in the genome of the wild relative relative to wheat is required. However, a polymorphic SNP between an allopolyploid species and wheat requires that each of the genomes of the wild relative carry the same polymorphism relative to the three genomes of wheat. As a result, the attrition rate for identifying polymorphic SNPs is much greater in allopolyploid wild relatives.

GISH has validated the genotyping work and significant numbers of introgressions have been indentified and characterised (Fig. 7). However, we are not presently able to determine whether we lack SNPs for parts of the genome. If this is the case, then we would not have been able to detect introgressions from these regions of the genome.

Previous results have suggested that the E genome shares the greatest level of homology with the D genome of wheat (Liu et al. 2007; Wang 2011; Hu et al. 2012). This was also suggested by the results of the synteny analysis in this work where the D genome was shown to have the highest number of significant BLASTS hits for the markers on the *Th. elongatum* genetic map (Fig. 2). It was therefore unexpected to find an almost equal number of recombination events between the E genome and the A and B genomes and the least number with the D genome (Fig. 8). The number of lines looked at with mc-GISH, however, was relatively small and thus more introgressions need to be studied. Much of the previous work has also been done on diploid accessions of *Th. elongatum* and thus greater clarity is required as to the genome composition of the higher ploidy levels. Good overall synteny is maintained between wheat and *Th. elongatum*, showing the close relationship of the two genomes. However, *Th. elongatum* does not carry a reciprocal 4A/5A/7B translocation that is observed in the A and B genomes of wheat (Devos et al. 1995).

**Fig. 9:**
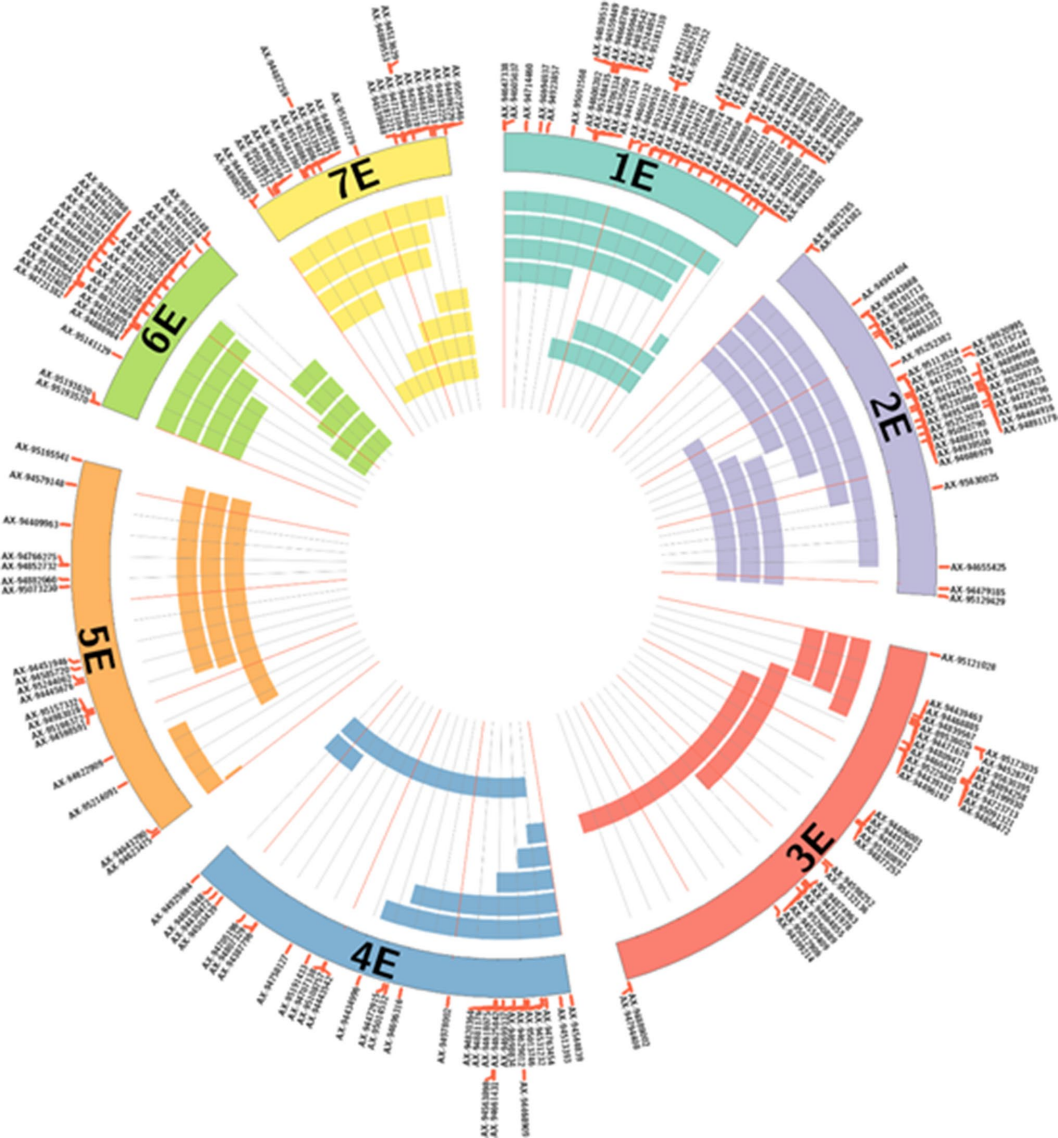
Graphical representation made using Circos showing the introgression sizes in panel plants from the wheat/*Th. elongatum* breeding programme. Each band in each linkage group represents a different individual line. Lines selected may contain whole chromosomes in other linkage groups. A total of 48 plants make up the panel

Most recombination events in wheat occur towards the distal regions of the chromosomes with large blocks in the pericentric regions experiencing only very low levels of recombination (Lukaszewski and Curtis 1993; Akhunov et al. 2003). However, in contrast to wheat, recombination in the interspecific wheat/*Th. elongatum* hybrids was not restricted to the distal regions of chromosomes, i.e. recombination was observed in both the distal and proximal regions of chromosomes (Fig. 8). It will be interesting to compare the recombination observed in the wheat/*Th. elongatum* hybrids with hybrids between wheat and other wild relatives, i.e. there is some evidence that mainly distal recombination occurs between wheat and *Haynaldia villosa* group 4 chromosome (Dai et al. 2020). A direct consequence of the localisation of chiasma to the distal regions of chromosomes in wheat is that genes located in the proximal regions of chromosomes will be inherited as unrecombined blocks, i.e. the generation of new allelic combinations will not occur in the proximal regions of chromosomes. Thus, the development of new allelic combinations for use in developing superior wheat varieties in breeding programmes will be limited to the genes located in the distal regions of chromosomes. Therefore, in order to generate new allelic combinations, a major focus of fundamental research in wheat is to shift recombination to the proximal regions of chromosomes. Thus, the observations described here demonstrate that the use of the wheat wild relative germplasm will provide an important means in manipulating and understanding the fundamental process of the position of recombination in wheat.

At present, work is underway using molecular markers complemented with cytogenetic analyses to select plants with single introgressions that represent the entire genome of *Th. elongatum* in overlapping segments (Fig. 9). After further rounds of self-fertilisation, a final panel will be composed of lines homozygous for different single introgressions and made available upon request.

## Electronic supplementary material

Below is the link to the electronic supplementary material.

Supplementary material 1 (DOCX 81 kb)
