# Supplementary material for: Exploiting the genome of Thinopyrum elongatum to expand the gene pool of hexaploid wheat
Source: Theor Appl Genet. 2020 Apr 20;133(7):2213–26. doi: 10.1007/s00122-020-03591-3 (PMC7311493; doi:10.1007/s00122-020-03591-3)
Supplement: Supplementary file 1 — Supplementary material 1 (DOCX 81 kb) [file 122_2020_3591_MOESM1_ESM.docx]

## Supplementary Table S1. The sequences of the 263 Axiom markers used to generate the genetic linkage map were used in a BLAST search (e-value cut-off of 1e-05) against the wheat genome IWGSC RefSeq v1.0 (Alaux *et al*., 2018; IWGSC *et al*., 2018) to obtain the corresponding physical positions of the top hit in the A, B and D genomes of wheat. Cells highlighted in yellow indicate the best BLAST hit for that marker.

| # | Marker ID | cM | Elong Chr | Wheat Chr | A BLAST pos | Wheat Chr | B BLAST pos | Wheat Chr | D BLAST pos |
| --- | --- | --- | --- | --- | --- | --- | --- | --- | --- |
| 1 | AX-94647338 | 0 | 1E | 1A | 1210273 | 1B | 1419791 | 1D | 2147569 |
| 2 | AX-94605037 | 0 | 1E |  |  | 1B | 8271108 | 1D | 6265880 |
| 3 | AX-94714460 | 1.749 | 1E | 1A | 7480848 |  |  | 1D | 6399538 |
| 4 | AX-94694937 | 2.909 | 1E |  |  | 1B | 8772906 | 1D | 6476995 |
| 5 | AX-94923857 | 3.485 | 1E | 1A | 11691168 | 1B | 15142424 |  |  |
| 6 | AX-95091568 | 5.532 | 1E | 1A | 26554647 | 1B | 40999694 | 1D | 25731691 |
| 7 | AX-95248435 | 7.282 | 1E | 1A | 30845354 |  |  | 1D | 32740064 |
| 8 | AX-94706334 | 7.282 | 1E | 1A | 32485071 | 1B | 52760447 | 1D | 34619013 |
| 9 | AX-94600202 | 7.282 | 1E | 1A | 48692194 | 1B | 69605659 | 1D | 49588712 |
| 10 | AX-94825050 | 7.282 | 1E | 1A | 52781502 | 1B | 87458634 | 1D | 53380946 |
| 11 | AX-94431524 | 7.282 | 1E | 1A | 53595738 | 1B | 90425378 | 1D | 55411516 |
| 12 | AX-94668789 | 7.282 | 1E | 1A | 58527735 | 1B | 96241391 | 1D | 60287855 |
| 13 | AX-94659645 | 7.282 | 1E | 1A | 60978182 | 1B | 100374718 | 1D | 63020896 |
| 14 | AX-94559449 | 7.282 | 1E | 1A | 67967070 | 1B | 110751408 | 1D | 69179557 |
| 15 | AX-94838542 | 7.282 | 1E | 1A | 98610732 | 1B | 132271340 | 1D | 80447489 |
| 16 | AX-94639519 | 7.282 | 1E |  |  | 1B | 156995645 | 1D | 77010121 |
| 17 | AX-95244854 | 7.282 | 1E | 1A | 110725875 | 1B | 158255152 | 1D | 108109856 |
| 18 | AX-95088840 | 7.282 | 1E | 1A | 114137804 | 1B | 167433722 | 1D | 110334716 |
| 19 | AX-94969312 | 7.282 | 1E | 1A | 126668338 | 1B | 177548863 | 1D | 116003162 |
| 20 | AX-95181310 | 7.282 | 1E | 1A | 156905284 | 1B | 206105620 | 1D | 131290910 |
| 21 | AX-94603132 | 10.233 | 1E | 1A | 226852803 | 1B | 249093668 |  |  |
| 22 | AX-94609516 | 10.233 | 1E | 1A | 257169006 | 1B | 296685411 | 1D | 203318416 |
| 23 | AX-95243397 | 11.1 | 1E | 1A | 258669282 | 1B | 299229609 | 1D | 204056097 |
| 24 | AX-94415591 | 11.967 | 1E | 1A | 260430639 | 1B | 299901346 | 1D | 204478355 |
| 25 | AX-94816469 | 11.967 | 1E | 1A | 260731103 | 1B | 300148101 | 1D | 204633724 |
| 26 | AX-94614192 | 11.967 | 1E | 1A | 363138230 | 1B | 320495507 | 1D | 250746175 |
| 27 | AX-94585755 | 11.967 | 1E | 1A | 304598636 | 1B | 333169017 | 1D | 240592901 |
| 28 | AX-95247252 | 11.967 | 1E | 1A | 325809094 | 1B | 357534128 | 1D | 254610784 |
| 29 | AX-94731169 | 11.967 | 1E | 1A | 319818263 | 1B | 363932366 | 1D | 250140005 |
| 30 | AX-95249741 | 12.834 | 1E | 1A | 362460722 | 1B | 391076809 | 1D | 290447089 |
| 31 | AX-94457686 | 13.701 | 1E | 1A | 365087871 | 1B | 394002829 | 1D | 293459188 |
| 32 | AX-95188674 | 14.277 | 1E | 1A | 369692522 | 1B | 399797117 | 1D | 297526295 |
| 33 | AX-94631754 | 15.437 | 1E | 1A | 376071765 | 1B | 406785441 | 1D | 301624918 |
| 34 | AX-94614812 | 15.437 | 1E | 1A | 376887720 | 1B | 408740709 | 1D | 302378115 |
| 35 | AX-94700816 | 15.437 | 1E | 1A | 376886974 | 1B | 408741455 | 1D | 302378861 |
| 36 | AX-94815097 | 15.437 | 1E | 1A | 376676515 | 1B | 409115644 | 1D | 302708755 |
| 37 | AX-95248891 | 15.437 | 1E | 1A | 398824941 | 1B | 429224799 | 1D | 317995967 |
| 38 | AX-94959080 | 15.437 | 1E | 1A | 406178144 | 1B | 434820942 | 1D | 322381706 |
| 39 | AX-95215437 | 16.304 | 1E | 1A | 408083406 | 1B | 441526965 | 1D | 327930543 |
| 40 | AX-94680423 | 17.463 | 1E | 1A | 425310094 |  |  | 1D | 328879677 |
| 41 | AX-94976931 | 17.463 | 1E | 1A | 429831685 | 1B | 446788614 | 1D | 332575456 |
| 42 | AX-94799746 | 17.463 | 1E | 1A | 432366172 | 1B | 450542432 | 1D | 335272744 |
| 43 | AX-94619761 | 17.463 | 1E | 1A | 442794282 | 1B | 460764002 | 1D | 343889191 |
| 44 | AX-94778702 | 17.463 | 1E | 1A | 462030245 | 1B | 485975385 | 1D | 363280574 |
| 45 | AX-95201195 | 18.04 | 1E | 1A | 463422741 | 1B | 487643693 | 1D | 364770883 |
| 46 | AX-94449858 | 18.907 | 1E |  |  | 1B | 488178189 | 1D | 365298603 |
| 47 | AX-94620619 | 18.907 | 1E | 1A | 463844802 | 1B | 488248170 | 1D | 365436461 |
| 48 | AX-94879529 | 18.907 | 1E | 1A | 466522659 | 1B | 491419715 | 1D | 367429627 |
| 49 | AX-94648380 | 18.907 | 1E |  |  | 1B | 510417130 |  |  |
| 50 | AX-94590777 | 19.194 | 1E | 1A | 499574210 | 1B | 543565949 | 1D | 404490127 |
| 51 | AX-94923890 | 19.194 | 1E | 1A | 500159956 | 1B | 544434677 | 1D | 405112632 |
| 52 | AX-94830058 | 19.194 | 1E | 1A | 502965851 | 1B | 547971666 | 1D | 407873726 |
| 53 | AX-94811680 | 20.353 | 1E | 1A | 502966099 | 1B | 547971913 | 1D | 407873974 |
| 54 | AX-94400160 | 20.353 | 1E | 1A | 509968062 | 1B | 557357542 | 1D | 413415744 |
| 55 | AX-94889522 | 20.353 | 1E | 1A | 514286748 | 1B | 564312270 | 1D | 418045571 |
| 56 | AX-94777625 | 20.93 | 1E | 1A | 517010379 | 1B | 569679627 | 1D | 421037416 |
| 57 | AX-94927609 | 20.93 | 1E | 1A | 517614136 | 1B | 570463526 | 1D | 422056065 |
| 58 | AX-94426657 | 20.93 | 1E | 1A | 520918061 | 1B | 575270950 | 1D | 426181095 |
| 59 | AX-94984326 | 20.93 | 1E | 1A | 521239246 | 1B | 575622537 | 1D | 426599424 |
| 60 | AX-95199064 | 21.217 | 1E | 1A | 539561033 | 1B | 603710539 | 1D | 444336901 |
| 61 | AX-95239366 | 21.217 | 1E | 1A | 539749370 | 1B | 604411040 | 1D | 444865694 |
| 62 | AX-94434557 | 21.217 | 1E | 1A | 545813011 | 1B | 620668263 | 1D | 451627063 |
| 63 | AX-94696169 | 21.505 | 1E | 1A | 551236186 | 1B | 631563476 | 1D | 460159035 |
| 64 | AX-94382911 | 21.505 | 1E | 1A | 560930651 | 1B | 646634917 | 1D | 468467843 |
| 65 | AX-94758797 | 21.505 | 1E | 1A | 563697182 | 1B | 652599796 | 1D | 470482367 |
| 66 | AX-95145266 | 22.081 | 1E | 1A | 574805966 | 1B | 666472243 | 1D | 478970113 |
| 67 | AX-94764182 | 22.081 | 1E | 1A | 577186739 | 1B | 668695987 | 1D | 481075003 |
| 68 | AX-94438392 | 22.081 | 1E | 1A | 587896667 | 1B | 681199649 | 1D | 488972853 |
| 69 | AX-94675785 | 0 | 2E | 2A | 16086112 | 2B | 24902468 |  |  |
| 70 | AX-94424382 | 0 | 2E | 2A | 16523810 |  |  |  |  |
| 71 | AX-94947404 | 5.764 | 2E | 2A | 18595090 | 2B | 29556642 | 2D | 17605335 |
| 72 | AX-94943668 | 6.923 | 2E | 2A | 24135836 |  |  | 2D | 22541774 |
| 73 | AX-95191713 | 6.923 | 2E | 2A | 42476155 | 2B | 65375492 | 2D | 37613883 |
| 74 | AX-94903195 | 6.923 | 2E | 2A | 42720363 | 2B | 66215037 | 2D | 37828636 |
| 75 | AX-95256835 | 8.083 | 2E |  |  | 2B | 74001772 | 2D | 46581121 |
| 76 | AX-94681135 | 8.659 | 2E | 2A | 50959627 | 2B | 75616460 | 2D | 47422524 |
| 77 | AX-94663017 | 8.947 | 2E | 2A | 62369345 | 2B | 97394507 | 2D | 62031868 |
| 78 | AX-95252382 | 11.594 | 2E |  |  | 2B | 105992938 | 2D | 69536387 |
| 79 | AX-95113524 | 12.754 | 2E | 2A | 102686603 | 2B | 154986086 | 2D | 106321792 |
| 80 | AX-95222525 | 12.754 | 2E |  |  | 2B | 166858639 |  |  |
| 81 | AX-94735763 | 12.754 | 2E | 2A | 119088248 | 2B | 168622110 | 2D | 117840249 |
| 82 | AX-95172933 | 12.754 | 2E | 2A | 176892574 | 2B | 225265109 | 2D | 172992426 |
| 83 | AX-94620995 | 12.754 | 2E | 2A | 209384458 | 2B | 250728558 | 2D | 194751887 |
| 84 | AX-95175724 | 13.33 | 2E | 2A | 284608601 |  |  |  |  |
| 85 | AX-94944759 | 14.197 | 2E | 2A | 456495939 | 2B | 410181825 | 2D | 344969517 |
| 86 | AX-95235860 | 14.485 | 2E | 2A | 530754282 | 2B | 464681719 | 2D | 392695843 |
| 87 | AX-94896956 | 14.772 | 2E | 2A | 550627212 | 2B | 482736359 | 2D | 406955669 |
| 88 | AX-94953488 | 15.348 | 2E | 2A | 574996961 | 2B | 507828716 | 2D | 430004718 |
| 89 | AX-94885008 | 15.348 | 2E | 2A | 577308122 | 2B | 510591634 | 2D | 431680330 |
| 90 | AX-95209735 | 15.348 | 2E | 2A | 582398277 | 2B | 517436565 | 2D | 439463097 |
| 91 | AX-94793623 | 15.348 | 2E | 2A | 584512391 | 2B | 518741377 | 2D | 440768654 |
| 92 | AX-95252073 | 15.636 | 2E | 2A | 584512592 | 2B | 518741578 | 2D | 440768855 |
| 93 | AX-95185447 | 15.636 | 2E | 2A | 584512705 | 2B | 518741691 | 2D | 440768973 |
| 94 | AX-94724796 | 15.923 | 2E | 2A | 590817254 | 2B | 526670405 | 2D | 446652662 |
| 95 | AX-94893293 | 15.923 | 2E | 2A | 602118038 | 2B | 539650983 | 2D | 458720262 |
| 96 | AX-94699666 | 15.923 | 2E | 2A | 615488238 | 2B | 553876523 | 2D | 473436854 |
| 97 | AX-95092790 | 16.499 | 2E | 2A | 619859620 | 2B | 559429751 | 2D | 477435470 |
| 98 | AX-94464916 | 16.787 | 2E | 2A | 631533800 |  |  | 2D | 485987719 |
| 99 | AX-94888719 | 17.074 | 2E | 2A | 631541058 | 2B | 570898764 | 2D | 485993834 |
| 100 | AX-94891179 | 17.074 | 2E | 2A | 634105236 | 2B | 574712815 | 2D | 490570476 |
| 101 | AX-95120881 | 17.074 | 2E | 2A | 637197415 | 2B | 576696201 | 2D | 491951990 |
| 102 | AX-94863536 | 17.074 | 2E | 2A | 665708465 | 2B | 612686744 | 2D | 520524075 |
| 103 | AX-94742957 | 17.074 | 2E | 2A | 671711934 | 2B | 622922141 | 2D | 526526324 |
| 104 | AX-95127627 | 17.074 | 2E |  |  | 2B | 636091036 | 2D | 534736147 |
| 105 | AX-94567662 | 17.361 | 2E | 2A | 704758812 | 2B | 675337578 | 2D | 564077736 |
| 106 | AX-94937792 | 17.361 | 2E | 2A | 706892535 | 2B | 680044621 | 2D | 567046192 |
| 107 | AX-94939500 | 17.649 | 2E | 2A | 716456182 | 2B | 695840381 | 2D | 578928999 |
| 108 | AX-94686979 | 18.225 | 2E | 2A | 738389432 | 2B | 740313390 | 2D | 606717489 |
| 109 | AX-95630025 | 22.096 | 2E | 2A | 738389309 | 2B | 740313513 | 2D | 606717366 |
| 110 | AX-94655425 | 28.507 | 2E |  |  | 2B | 743329373 | 2D | 609244095 |
| 111 | AX-94479185 | 30.257 | 2E | 2A | 744794363 | 2B | 748432547 | 2D | 613107237 |
| 112 | AX-95129429 | 30.833 | 2E | 2A | 768627752 | 2B | 793348942 | 2D | 643776110 |
| 113 | AX-95121028 | 0 | 3E | 3A | 23275445 | 3B | 18784942 | 3D | 13663694 |
| 114 | AX-94839567 | 5.125 | 3E | 3A | 35050454 | 3B | 41909723 | 3D | 25976096 |
| 115 | AX-89536025 | 5.412 | 3E | 3A | 61219798 | 3B | 76407963 | 3D | 48808264 |
| 116 | AX-94468885 | 5.412 | 3E | 3A | 102273250 | 3B | 133255193 | 3D | 85250955 |
| 117 | AX-94471678 | 5.412 | 3E | 5B | 211148109 | 3B | 149835669 |  |  |
| 118 | AX-94809471 | 5.412 | 3E | 3A | 108376379 | 3B | 162292144 | 3D | 112690769 |
| 119 | AX-94439463 | 5.7 | 3E | 3A | 112954004 | 3B | 168257540 | 3D | 116035670 |
| 120 | AX-95173035 | 5.7 | 3E | 3A | 127970162 | 3B | 171741648 | 3D | 121533327 |
| 121 | AX-94528741 | 5.7 | 3E | 3A | 159836014 | 3B | 202188233 | 3D | 141410294 |
| 122 | AX-94604377 | 5.7 | 3E | 7B | 603948740 | 4B | 428699960 |  |  |
| 123 | AX-95225885 | 7.449 | 3E | 3A | 428078701 | 3B | 413597365 | 3D | 317228537 |
| 124 | AX-94439183 | 8.026 | 3E | 3A | 467153151 | 3B | 446597858 | 3D | 348165315 |
| 125 | AX-94496167 | 8.026 | 3E | 3A | 477428563 | 3B | 461463966 | 3D | 358185046 |
| 126 | AX-95199930 | 8.026 | 3E | 3A | 479100548 | 3B | 464482431 | 3D | 359638978 |
| 127 | AX-94723713 | 8.026 | 3E |  |  | 3B | 486036357 | 3D | 373609060 |
| 128 | AX-94894258 | 8.026 | 3E | 3A | 538536933 | 3B | 539236747 | 3D | 414773702 |
| 129 | AX-95091321 | 8.026 | 3E | 3A | 541967464 | 3B | 544008627 | 3D | 419523839 |
| 130 | AX-95630395 | 8.026 | 3E |  |  | 3B | 577121912 | 3D | 441199877 |
| 131 | AX-94856472 | 8.026 | 3E | 3A | 585023099 | 3B | 580470715 | 3D | 444067165 |
| 132 | AX-94931831 | 14.764 | 3E | 3A | 598563624 | 3B | 600870593 | 3D | 455886714 |
| 133 | AX-95180897 | 14.764 | 3E |  |  | 3B | 620789922 |  |  |
| 134 | AX-94497957 | 14.764 | 3E | 3A | 610525422 | 3B | 620790014 | 3D | 468663280 |
| 135 | AX-94877257 | 14.764 | 3E | 3A | 611702729 | 3B | 621657243 | 3D | 469162181 |
| 136 | AX-94406001 | 14.764 | 3E | 3A | 616281010 | 3B | 628805002 | 3D | 473830108 |
| 137 | AX-94598252 | 18.945 | 3E | 3A | 616288208 | 3B | 628813209 | 3D | 473837317 |
| 138 | AX-95132136 | 19.232 | 3E | 3A | 631579378 | 3B | 649309291 | 3D | 487818076 |
| 139 | AX-94741978 | 21.279 | 3E | 3A | 631584230 | 3B | 649314382 | 3D | 487822880 |
| 140 | AX-94664855 | 21.279 | 3E | 3A | 638062444 | 3B | 654367108 | 3D | 496751915 |
| 141 | AX-94874963 | 21.279 | 3E |  |  | 3B | 669035708 | 3D | 508191324 |
| 142 | AX-95260889 | 21.567 | 3E | 3A | 662395465 | 3B | 696395050 | 3D | 527673517 |
| 143 | AX-94555409 | 21.567 | 3E | 3A | 674754383 | 3B | 714385019 | 3D | 539914485 |
| 144 | AX-95012906 | 22.143 | 3E | 3A | 692477247 | 3B | 736650521 | 3D | 555150826 |
| 145 | AX-94399214 | 24.49 | 3E |  |  | 3B | 780986653 | 3D | 585923134 |
| 146 | AX-94889002 | 37.881 | 3E | 3A | 749332842 | 3B | 823378265 | 3D | 613703568 |
| 147 | AX-94794408 | 37.881 | 3E | 3A | 749332834 | 3B | 823378273 | 3D | 613703560 |
| 148 | AX-94544839 | 0 | 4E | 4A | 595376411 | 4B | 14340972 | 4D | 7902539 |
| 149 | AX-94513393 | 0.867 | 4E | 4A | 584124816 | 4B | 28143983 | 4D | 16246414 |
| 150 | AX-94763454 | 2.027 | 4E | 4A | 584068062 | 4B | 28192918 | 4D | 16355938 |
| 151 | AX-94531232 | 2.314 | 4E | 2A | 102327462 | 2B | 154720797 | 2D | 103166881 |
| 152 | AX-95013746 | 2.314 | 4E | 4A | 450339958 | 4B | 182239845 | 4D | 119748632 |
| 153 | AX-94629012 | 2.314 | 4E | 4A | 438617231 |  |  | 4D | 128913370 |
| 154 | AX-94698834 | 3.473 | 4E | 4A | 329182808 | 4B | 358480571 | 4D | 281298949 |
| 155 | AX-94699337 | 3.761 | 4E | 4A | 182923401 | 4B | 373762169 | 4D | 300281422 |
| 156 | AX-94468909 | 4.048 | 4E | 4A | 181621592 | 4B | 374729541 | 4D | 301021779 |
| 157 | AX-94625842 | 4.624 | 4E | 4A | 175412184 | 4B | 378767724 | 4D | 303835881 |
| 158 | AX-94618075 | 5.492 | 4E | 4A | 150229847 | 4B | 398559401 | 4D | 322674085 |
| 159 | AX-94881176 | 6.068 | 4E | 4A | 150230370 | 4B | 398559933 | 4D | 322673558 |
| 160 | AX-94661431 | 6.068 | 4E | 4A | 153252145 | 4B | 400695469 | 4D | 320037277 |
| 161 | AX-94820364 | 6.068 | 4E | 4A | 136796386 | 4B | 414798099 | 4D | 336537822 |
| 162 | AX-94563898 | 6.068 | 4E | 4A | 135615014 | 4B | 416305627 | 4D | 337152532 |
| 163 | AX-94978002 | 9.939 | 4E | 4A | 132992597 | 4B | 418520276 | 4D | 339077665 |
| 164 | AX-94696316 | 13.81 | 4E | 4A | 117280140 | 4B | 428467206 | 4D | 348310679 |
| 165 | AX-95014532 | 14.969 | 4E | 4A | 116471600 | 4B | 428970973 | 4D | 348802338 |
| 166 | AX-94472915 | 15.257 | 4E | 4A | 114587563 | 4B | 433646453 | 4D | 350501930 |
| 167 | AX-94434996 | 17.304 | 4E | 4A | 103717374 | 4B | 446515863 | 4D | 361036550 |
| 168 | AX-95108757 | 20.254 | 4E | 4A | 98597281 | 4B | 450917795 | 4D | 366326040 |
| 169 | AX-94707138 | 20.254 | 4E | 4A | 87972681 | 4B | 463717793 | 4D | 377852800 |
| 170 | AX-94443542 | 20.254 | 4E | 4A | 87412354 | 4B | 464160632 | 4D | 378262910 |
| 171 | AX-95191433 | 21.708 | 4E | 4A | 59482630 | 4B | 497552601 | 4D | 402209210 |
| 172 | AX-94758127 | 23.458 | 4E | 4A | 48531795 | 4B | 511159432 | 4D | 414995354 |
| 173 | AX-94387796 | 26.713 | 4E | 4A | 16191593 |  |  | 4D | 450509926 |
| 174 | AX-94807329 | 26.713 | 4E | 4A | 11811427 | 4B | 569630638 | 4D | 455746040 |
| 175 | AX-94705196 | 27.873 | 4E |  |  | 4B | 600922067 | 4D | 476870248 |
| 176 | AX-94503439 | 30.219 | 4E | 4A | 684661325 | 4B | 604204008 | 4D | 478033278 |
| 177 | AX-94430472 | 30.795 | 4E | 5A | 664781708 | 4B | 616274543 | 4D | 484358277 |
| 178 | AX-94681948 | 31.372 | 4E | 5A | 680209821 |  |  | 4D | 499451971 |
| 179 | AX-94925964 | 32.825 | 4E | 5A | 698359181 | 4B | 657274162 | 4D | 509480988 |
| 180 | AX-94623475 | 0 | 5E |  |  | 5B | 16033800 | 5D | 22441122 |
| 181 | AX-94643290 | 0.287 | 5E | 5A | 79284406 | 5B | 92233272 |  |  |
| 182 | AX-95214091 | 4.781 | 5E | 5A | 92174101 | 5B | 103317935 | 5D | 97037155 |
| 183 | AX-94622909 | 7.128 | 5E | 5A | 173115023 | 5B | 163089990 | 5D | 184039993 |
| 184 | AX-95166372 | 10.999 | 5E |  |  | 5B | 196269757 | 5D | 193131241 |
| 185 | AX-94983016 | 10.999 | 5E | 5A | 227707889 | 5B | 209650704 | 5D | 198952444 |
| 186 | AX-94598591 | 11.286 | 5E | 5A | 307349385 | 5B | 254573105 | 5D | 228776744 |
| 187 | AX-95157332 | 11.286 | 5E | 5A | 309100178 | 5B | 255550390 | 5D | 242966391 |
| 188 | AX-94445676 | 13.633 | 5E |  |  |  |  | 5D | 313101557 |
| 189 | AX-95244062 | 13.92 | 5E | 5A | 466986747 | 5B | 432086465 | 5D | 365325295 |
| 190 | AX-94585720 | 14.787 | 5E | 5A | 485203212 | 5B | 461137594 | 5D | 384344057 |
| 191 | AX-94451946 | 15.363 | 5E | 5A | 494870808 | 5B | 471859840 | 5D | 391737025 |
| 192 | AX-95073230 | 21.45 | 5E |  |  | 5B | 539289191 | 5D | 443008712 |
| 193 | AX-94882660 | 22.026 | 5E | 5A | 591318697 | 5B | 578367874 | 5D | 471047408 |
| 194 | AX-94852732 | 23.186 | 5E | 5A | 595037854 | 5B | 582975465 | 5D | 475575769 |
| 195 | AX-94766275 | 23.186 | 5E | 5A | 598851795 | 5B | 588500844 | 5D | 480351898 |
| 196 | AX-94409963 | 26.441 | 5E | 5A | 599218908 |  |  | 5D | 480476392 |
| 197 | AX-94579148 | 29.697 | 5E | 4A | 611196985 |  |  |  |  |
| 198 | AX-95165541 | 31.744 | 5E | 4A | 640003384 | 5B | 671356286 | 5D | 532247938 |
| 199 | AX-95193570 | 0 | 6E |  |  |  |  | 6D | 3917370 |
| 200 | AX-95191620 | 0 | 6E | 6A | 34982719 | 6B | 64233881 |  |  |
| 201 | AX-95141129 | 4.182 | 6E | 6A | 79268695 | 6B | 135859976 | 6D | 62115302 |
| 202 | AX-94784805 | 6.829 | 6E | 6A | 84922633 | 6B | 142229560 | 6D | 67377364 |
| 203 | AX-86167869 | 6.829 | 6E | 6A | 90032550 | 6B | 147961353 | 6D | 73587857 |
| 204 | AX-94555013 | 6.829 | 6E | 6A | 102106288 | 6B | 161588687 | 6D | 85040070 |
| 205 | AX-95218218 | 6.829 | 6E | 6A | 104052449 | 6B | 163969106 | 6D | 86546850 |
| 206 | AX-94888984 | 7.117 | 6E | 6A | 107113330 | 6B | 169318510 | 6D | 89004173 |
| 207 | AX-95183306 | 7.117 | 6E | 6A | 110348253 | 6B | 175726894 | 6D | 91567850 |
| 208 | AX-95143205 | 7.117 | 6E | 6A | 123536433 | 6B | 188189414 | 6D | 102467654 |
| 209 | AX-94880647 | 7.117 | 6E | 6A | 135245170 | 6B | 199763563 | 6D | 110882126 |
| 210 | AX-94932402 | 7.117 | 6E | 6A | 135247517 | 6B | 199765840 | 6D | 110884476 |
| 211 | AX-94824033 | 7.404 | 6E | 6A | 137375714 | 6B | 201408229 | 6D | 112429665 |
| 212 | AX-94721382 | 7.404 | 6E | 5A | 524244857 | 5B | 496526921 | 5D | 412365058 |
| 213 | AX-94712565 | 8.271 | 6E | 6A | 171456070 | 6B | 229724853 | 6D | 132410006 |
| 214 | AX-94876114 | 8.271 | 6E | 6A | 258141313 | 6B | 309746964 | 6D | 163836156 |
| 215 | AX-94975749 | 8.558 | 6E | 6A | 356971915 | 6B | 347226740 | 6D | 262752104 |
| 216 | AX-94666942 | 8.558 | 6E |  |  | 6B | 362872739 | 6D | 189415635 |
| 217 | AX-95193304 | 9.135 | 6E | 6A | 276310378 |  |  | 6D | 206550098 |
| 218 | AX-94748397 | 9.422 | 6E | 6A | 431175705 | 6B | 453928382 | 6D | 291960047 |
| 219 | AX-94872125 | 9.998 | 6E | 6A | 435774967 | 6B | 449767324 | 6D | 289217470 |
| 220 | AX-94536363 | 9.998 | 6E |  |  |  |  | 6D | 290319933 |
| 221 | AX-95252343 | 9.998 | 6E | 6A | 421771087 | 6B | 461263174 | 6D | 296199685 |
| 222 | AX-94407381 | 10.866 | 6E | 6A | 410893883 | 6B | 470129793 | 6D | 304288987 |
| 223 | AX-94419941 | 10.866 | 6E | 6A | 409091513 | 6B | 471230084 | 6D | 305358871 |
| 224 | AX-94946469 | 10.866 | 6E | 6A | 441499976 | 6B | 473064761 | 6D | 306137963 |
| 225 | AX-94562108 | 10.866 | 6E | 6A | 454939689 | 6B | 506723185 | 6D | 317474276 |
| 226 | AX-94541273 | 10.866 | 6E | 6A | 499645351 | 6B | 539054761 | 6D | 357567621 |
| 227 | AX-94761183 | 10.866 | 6E | 6A | 503767256 | 6B | 545219370 | 6D | 363060458 |
| 228 | AX-94664517 | 10.866 | 6E | 6A | 517664625 | 6B | 562558813 | 6D | 375811144 |
| 229 | AX-94495972 | 10.866 | 6E | 6A | 522226196 | 6B | 568256903 | 6D | 380799454 |
| 230 | AX-94956915 | 10.866 | 6E | 6A | 525993962 | 6B | 574021945 | 6D | 383746882 |
| 231 | AX-94611381 | 11.153 | 6E | 6A | 528990120 | 6B | 574627020 | 6D | 384362351 |
| 232 | AX-94797868 | 11.153 | 6E | 6A | 568494582 | 6B | 639147632 | 6D | 423788306 |
| 233 | AX-95130177 | 11.729 | 6E | 6A | 573508327 | 6B | 645549279 | 6D | 428923479 |
| 234 | AX-94532804 | 13.183 | 6E | 6A | 574479728 | 6B | 646629800 | 6D | 429311494 |
| 235 | AX-95192178 | 14.932 | 6E | 6A | 585196836 | 6B | 660676196 | 6D | 436887495 |
| 236 | AX-94768784 | 15.799 | 6E | 6A | 602706285 | 6B | 693092765 | 6D | 455886116 |
| 237 | AX-95142148 | 15.799 | 6E | 6A | 605103946 | 6B | 696993294 | 6D | 458529647 |
| 238 | AX-95072546 | 0 | 7E | 7A | 700637432 | 7B | 699804384 | 7D | 610780142 |
| 239 | AX-94699229 | 0 | 7E | 7A | 644060822 | 7B | 607605123 | 7D | 559416280 |
| 240 | AX-94938225 | 1.749 | 7E | 7A | 625566923 |  |  | 7D | 543686062 |
| 241 | AX-95081313 | 2.037 | 7E | 7A | 612039478 | 7B | 572007391 | 7D | 531576385 |
| 242 | AX-94513629 | 2.037 | 7E |  |  | 7B | 565881249 | 7D | 528089422 |
| 243 | AX-94889553 | 2.324 | 7E | 7A | 596642613 | 7B | 553598507 | 7D | 520357632 |
| 244 | AX-94468312 | 4.671 | 7E | 7A | 514936877 | 7B | 505541545 | 7D | 479697500 |
| 245 | AX-94701218 | 4.671 | 7E | 7A | 514936944 | 7B | 505541456 | 7D | 479697411 |
| 246 | AX-94539848 | 4.671 | 7E |  |  |  |  | 7D | 457971005 |
| 247 | AX-94649688 | 4.958 | 7E | 7A | 501574700 | 7B | 448702041 | 7D | 431209283 |
| 248 | AX-94712104 | 5.534 | 7E | 7A | 514597909 | 7B | 434812371 | 7D | 418918545 |
| 249 | AX-95181221 | 6.111 | 7E |  |  | 7B | 388139516 | 7D | 391532758 |
| 250 | AX-95107279 | 6.111 | 7E |  |  |  |  | 7D | 388593269 |
| 251 | AX-94487259 | 9.366 | 7E | 7A | 427745960 | 7B | 374698680 | 7D | 379525343 |
| 252 | AX-94385484 | 12.622 | 7E | 7A | 355617090 | 7B | 342102812 | 7D | 301977434 |
| 253 | AX-94801375 | 13.198 | 7E | 7A | 294339315 | 7B | 248522263 | 7D | 268501736 |
| 254 | AX-94533947 | 13.198 | 7E | 7A | 288853936 | 7B | 245043371 | 7D | 264698879 |
| 255 | AX-95237806 | 13.486 | 7E | 7A | 187032176 | 7B | 152769519 | 7D | 183743168 |
| 256 | AX-95140865 | 13.486 | 7E |  |  | 7B | 152757313 |  |  |
| 257 | AX-94581390 | 13.486 | 7E | 7A | 182544195 | 7B | 144634324 | 7D | 178558924 |
| 258 | AX-94500577 | 13.486 | 7E | 7A | 122460833 | 7B | 79283844 | 7D | 118108367 |
| 259 | AX-94905259 | 13.486 | 7E | 7A | 92965486 | 7B | 42333332 | 7D | 90954559 |
| 260 | AX-95018673 | 14.062 | 7E | 7A | 82142933 | 7B | 26748291 | 7D | 79490036 |
| 261 | AX-94758772 | 15.516 | 7E | 7A | 58791942 |  |  | 7D | 55029019 |
| 262 | AX-94456809 | 15.803 | 7E | 7A | 42076676 | 4A | 650044562 | 7D | 42649821 |
| 263 | AX-94900297 | 16.67 | 7E | 7A | 979619 | 4A | 724790450 | 7D | 13006579 |
